# A Deterministic Spectral‐Design Method for Guided‐Mode Resonances

**DOI:** 10.1002/nap2.70181

**Published:** 2026-06-26

**Authors:** Joong Hyun Pyo, Yu Sung Choi, Robert Magnusson, Jae Woong Yoon

**Affiliations:** ^1^ Department of Physics Hanyang University Seoul Korea; ^2^ Department of Electrical Engineering University of Texas Arlington Texas USA

## Abstract

Resonance frequency and linewidth are key metrics for guided‐mode resonance (GMR) devices and their independent control is important for practical applications. Trial–solution‐based approaches have been used to obtain appropriate element designs with desired key metrics. In this paper, we propose a dual‐ridge compound unit‐cell structure for fully independent key‐metric control in a deterministic manner. We derive a concise design‐parametric search space that analytically relates the key GMR metrics with the unit‐cell geometry without undesirable complications which are inherent in the previous approaches. On this basis, we demonstrate completely independent control on the multiple key GMR metrics. In addition, our proposed approach describes how non‐Hermitian and topological features such as Fermi arcs and exceptional points emerge in GMR spectra.

## Graphical abstract

Resonance frequency and linewidth are key metrics for guided‐mode resonance devices and their independent control is important for practical applications. We propose a fully analytic deterministic design method for independently obtaining desired target metrics of guided‐mode resonances in dual‐ridge grating waveguide structures.

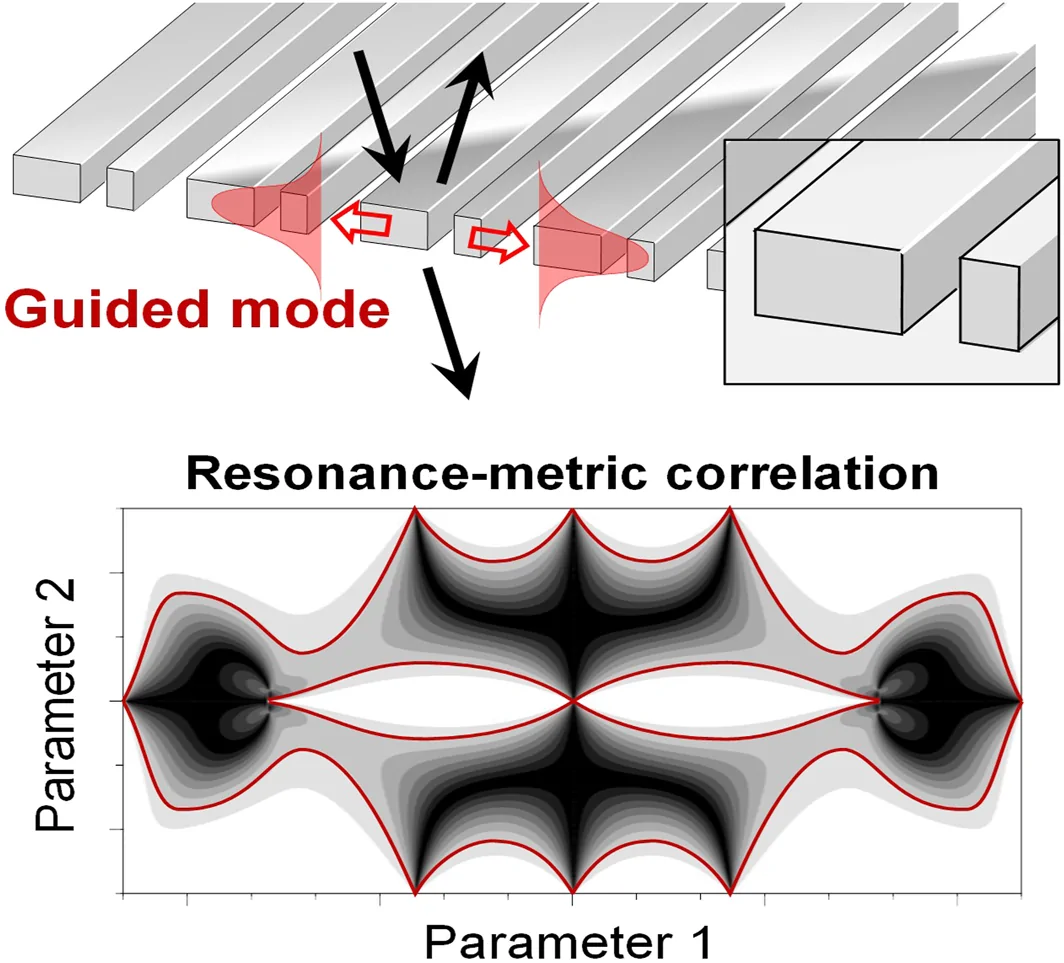

## 1 Introduction

Guided mode resonances (GMR) produce versatile spectral features for optical filters, sensors, lasers, and many others [1, 2, 3, 4]. In particular, GMRs around the second‐order band gap condition are of interest because they produce the strongest spectral expressions associated with total reflection or transmission peaks [5, 6, 7, 8, 9], symmetry‐protected bound states in the continuum (BIC) [10, 11], and topological edge states near normal‐incidence condition [12, 13, 14, 15, 16]. Key metrics for GMR properties in this spectral region are band gap center frequency *ω*_c_, band gap width *ω*_g_, and resonance linewidth Δ*ω*. Optimizing a GMR element design reduces to a multi‐dimensional parametric search problem finding unit‐cell designs for desired metrics {*ω*_c_, *ω*_g_, Δ*ω*}.

GMR elements based on a single‐ridge unit cell have been widely used for its structural simplicity and subsequent advantages in design and experiments. In this case, a major design parameter is relative ridge width to the grating period, which is often referred to as fill factor *F*. *F* simultaneously controls key metrics *ω*_c_, *ω*_g_, and Δ*ω* in general. Independent control of these metrics is important in many applications in practice and it demands additional tuning parameters such as ridge height *h* and layer thicknesses *t*_*n*_ because the number of design‐parametric degrees of freedom should be fundamentally larger than the number of desired metrics. Nevertheless, such parametric tuning often fails to deliver an optimal design because respective impacts of *F*, *h*, and *t*_*n*_ on *ω*_g_ and Δ*ω* are nontrivial in general.

Toward this end, compound unit‐cell designs including multiple grating ridges may provide an efficient approach to the independent control as multiple fill factors and relative positions of ridges within the unit cell are available for additional design‐parametric degrees of freedom for directly controlling diffractive‐coupling amplitudes prescribed for the target metrics. They have been exploited as an efficient structure for spectral filters [17] and quasi‐bound states in the continuum (quasi‐BICs) [18, 19]. In those works, high‐*Q* resonances are obtained by breaking the mirror symmetry of a unit cell and subsequent modification of radiation properties of band‐edge states. Although delicate linewidth control methods were provided on such bases, a comprehensive systematic approach for independent resonance metric tuning has not been established, in spite of its immediate impact on GMR element development.

In this paper, we provide a comprehensive treatment of dual‐ridge compound unit‐cell structures and demonstrate how one can obtain completely independent control on the key GMR metrics. We utilize a coupled‐mode model for GMRs in our cases of interest. The model yields closed‐form expressions for *ω*_g_ and Δ*ω* in terms of fill factors and relative positions of two grating ridges. These expressions reveal a deterministic method of independently controlling *ω*_g_ and Δ*ω*. We perform rigorous numerical analysis and it quantitatively confirms the feasibility of our proposed approach.

## 2 Simplified Model Description

We start our argument with a simplified model of a transverse‐electric (TE) GMR. The model is based on the coupled‐mode theory (CMT) proposed by Kazarinov and Henry [20]. We consider a planar‐mount GMR structure and geometry as shown in Figure 1a. In this geometry, the CMT leads to an eigenvalue problem as follows:

(1) $$H|\psi \rangle =\left(\omega -\omega_{B}\right)|\psi \rangle ,$$

for GMR state |*ψ*〉 = [*a*_+_ *a*_−_]^T^ with *a*_±_ denoting amplitude of guided mode heading towards ± *x* direction (same sign in the same order). Here, *ω*_B_ is frequency of the second‐order Bragg condition for the guided mode and the effective Hamiltonian is given by the following equation:

(2) $$H=\left[\begin{array}{cc} vk+\delta & m_{+}\\ m_{-} & -vk+\delta \end{array}\right].$$

**FIGURE 1:**
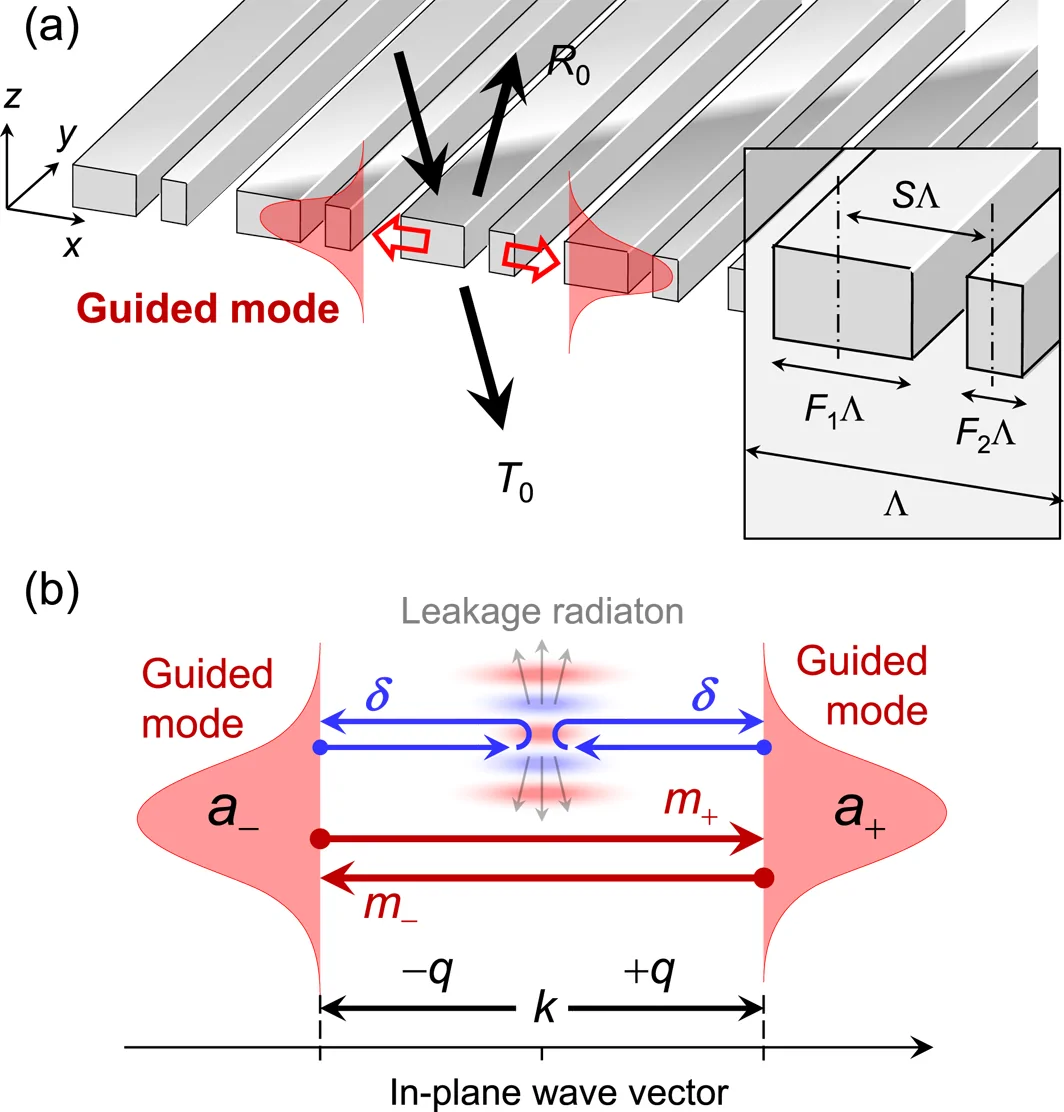
GMR in dual‐ridge compound unit‐cell structure. (a) Schematic of a unit‐cell design. (b) Guided‐mode interaction configuration on the wave‐vector domain by the effective Hamiltonian in Equation (2).

Here, *v* is group speed of the guided mode and *k* is Bloch wave vector in *x* axis. The guided mode considered here is TE_0_ mode that couples with the far‐field planewave through the first‐order diffraction. In such a condition, the structure produces a GMR at phase‐matching condition *k* ± 2π/Λ = *β*, where *β* denotes the propagation constant of TE_0_ guided mode. The effective Hamiltonian describes guided mode interaction by a configuration illustrated in Figure 1b. The two counter‐propagating guided modes are coupled to each other through the second‐order Bragg reflection at rate *m*_±_, as indicated by red arrows. This inter‐guided‐mode coupling results in standing‐wave normal modes which lead to band gap opening. Therefore, *m*_±_ is related to band gap width *ω*_g_ and the linewidth difference between the two bands.

Another important second‐order diffraction process is self‐coupling by the two consecutive first‐order diffraction events at rate *δ*, as indicated by blue arrows. In the self‐coupling process, the diffracted field from the guided mode transits through the zero‐order field of the leakage radiation. Therefore, *δ* is associated with radiation‐decay linewidth Δ*ω*.

Eigenstate solutions of Equation (1) represent normal modes that eventually build up as resonant states under time‐harmonic planewave excitations at their respective characteristic frequencies, that is, the eigenvalues of Equation (1) given by the following equation:

(3) $$\omega_{\pm }=\omega_{B}+\delta \pm \sqrt{v^{2}k^{2}+m_{+}m_{-}}.$$

This eigenvalue expression immediately yields band‐gap center frequency *ω*_c_, band gap width *ω*_g_, and line width Δ*ω*_±_ as follows:

(4) $$\omega_{c}=\frac{1}{2}\text{Re}\left(\omega_{+}+\omega_{-}\right)=\omega_{B}+\text{Re}\left(\delta \right),$$

(5) $$\omega_{g}=\text{Re}\left(\omega_{+}-\omega_{-}\right)=2\text{Re}\left(\sqrt{m_{+}m_{-}}\right),$$

(6) $$\Delta \omega_{\pm }=\text{Im}\left(\omega_{\pm }\right)=\text{Im}\left(\delta \pm \sqrt{m_{+}m_{-}+v^{2}k^{2}}\right).$$

Consequently, the independent control of these metrics requires appropriate tuning of Bragg reflection rate *m*_±_ and self‐coupling rate *δ* by means of the unit cell design.

Parameters *m*_±_ and *δ* are directly related to the unit‐cell geometry and we can make use of it for our design strategies independently controlling band gap width and linewidth. They are given in terms of Fourier transform {*ε*_*n*_} of the dielectric function in the grating region as follows:

(7) $$m_{\pm }={\varepsilon_{\pm 1}}^{2}\kappa_{1}-\varepsilon_{\pm 2}\kappa_{2},$$

(8) $$\delta =\varepsilon_{+1}\varepsilon_{-1}\kappa_{1},$$

where *κ*_1_ and *κ*_2_ are diffraction‐rate coefficients for the two consecutive first‐order diffraction and one second‐order diffraction of the guided mode, and *ε*_*n*_ denotes Fourier coefficient of the relative electric permittivity distribution in the grating layer at the *n*th harmonic order. Detailed expressions for these coefficients are provided in our previous work [21]. Briefly explaining the mathematical procedures leading to Equations (7) and (8), the total field is decomposed into the zero‐order harmonic leakage‐radiation and first‐order harmonic guided‐mode components and the coupling between these two field components are mathematically described by plugging them into the electromagnetic Helmholtz equation. This treatment leads to the effective Hamiltonian in Equation (2) as well as the diffractive coupling constant expressions in Equations (7) and (8). *m*_±_ in Equation (7) describes mutual diffractive coupling between the two counter‐propagating guided modes through second‐order diffraction processes, as indicated in Figure 1b. The dominant second‐order diffraction processes are two consecutive events of first‐order diffraction each driven by *ε*_±1_ and a single event of second‐order diffraction driven by *ε*_±2_. Therefore, the righthand‐side of Equation (7) accordingly consists of the two terms involving *ε*_±1_^2^ and *ε*_±2_. *δ* in Equation (8) represents self‐diffractive coupling indicated in Figure 1b. It is driven by two consecutive first‐order diffraction events, the first one in the backward direction and the second one in the forward direction with respect to the associated guided mode. Therefore, it contains the product *ε*_+1_ *ε*_−1_. In the argument hereafter, we assume lossless media for the entire structure and thereby {*ε*_*n*_} is subject to the conjugation symmetry, that is, *ε*_−*n*_ = *ε*_+n_*.

Equations (7) and (8) reveal the reason why the independent control of *ω*_c_, *ω*_g_, and Δ*ω*_±_ frequently becomes nontrivial for single‐ridge unit‐cell designs in which {*ε*_*n*_} is determined by the following equation:

(9) $$\varepsilon_{n\neq 0}=\Delta \varepsilon \;F\;\text{sinc}\left(nF\right),$$

where Δ*ε* is dielectric‐constant difference *ε*_high_ − *ε*_low_ in the grating region, Obviously, *m*_±_ and *δ* simultaneously change with *F* inevitably and so do *ω*_c_, *ω*_g_, and Δ*ω*_±_. Moreover, change in *F* alters average dielectric constant *ε*_0_ = *Fε*_high_ + (1 − *F*)*ε*_low_ which determines guided‐mode wave function and pertaining quantities including its propagation constant, Bragg‐reflection frequency *ω*_B_, group speed *v*, and diffraction rate coefficients *κ*_1_ and *κ*_2_ as well as further simultaneous changes in *m*_±_ and *δ*. These concurrent changes give rise to unavoidable complications in the structure optimization for desired key metrics.

Such complications can be avoided in dual‐ridge compound unit–cell structures in Figure 1a. The two ridges are characterized by their center‐to‐center spacing factor *S* and individual fill factors *F*_1_ and *F*_2_. These parameters form three‐dimensional (3D) search space {*F*_1_, *F*_2_, *S*} and thereby provide the enough number of degrees of design freedom for potentially independent control of *ω*_g_ and Δ*ω*_±_. In this 3D search space, we can configure a plausible subspace that avoids the complication arising from strong fill‐factor dependence of *ε*_0_ for the single‐ridge unit–cell structures. Such subspace is obtained by restricting net fill factor *F* = *F*_1_ + *F*_2_ to be constant. Any changes in *F*_1_, *F*_2_, and *S* within the constant‐F subspace do not alter average dielectric constant *ε*_0_, minimizing undesirable modifications of interaction coefficients associated with it. In particular, the subspace keeps *ω*_B_, *v*, *κ*_1_, and *κ*_2_ being constant within the CMT model. Considering *ω*_B_ >> |*δ*| for sufficiently strong GMRs in general, we can reasonably assume that the band‐gap‐center frequency *ω*_c_ in Equation (4) is practically constant over this parametric subspace. Subsequently, we focus on the independent control of *ω*_g_ and Δ*ω*_±_ hereafter.

Making convenient use of the constant‐F subspace, we define the fill‐factor difference *χ* = *F*_1_ − *F*_2_ that sets fill factors by *F*_1_ = (*F*−*χ*)/2 and *F*_2_ = (*F*+*χ*)/2. Then, parametric subspace {*χ*, *S*} within range {[−*F*, +*F*], [*F*/2, 1 − *F*/2]} is our geometrically accessible constant‐F search space which we will use hereafter. Note that this parametric search space is a plane with its center at (*χ*, *S*) = (0, 1/2) and spans of (Δ*χ*, Δ*S*) = (2*F*, 1 − *F*). It is worth noting in this search space that a grating design reduces to a single‐ridge unit–cell structure at special locations. The grating design at the center (0, 1/2) is a half‐period (Λ → Λ/2) single‐ridge unit‐cell structure because of the mirror symmetry. On the outer boundaries at *χ* = ±F or *S* = [1 ± (1 − F)]/2, the design structure becomes a right‐period (Λ → Λ) single‐ridge unit–cell structure because the two ridges merge into a single ridge. In addition, the grating design is mirror‐symmetric with respect to *y*–*z* plane along two vertical lines at *χ* = 0 and *S* = 1/2.

Distributions of *ω*_g_ and Δ*ω*_±_ on *χ*‐*S* plane can be conveniently analyzed with closed‐form expressions. The real and imaginary parts of Fourier coefficient {*ε*_*n*_} take explicit expressions as follows:

(10) $$\text{Re}\left(\varepsilon_{n\neq 0}\right)=\frac{2\Delta \varepsilon }{n\pi }\;\sin\left(\frac{n\pi F}{2}\right)\cos\left(\frac{n\pi \chi }{2}\right)\cos\left(n\pi S\right),$$

(11) $$\text{Im}\left(\varepsilon_{n\neq 0}\right)=\frac{2\Delta \varepsilon }{n\pi }\;\cos\left(\frac{n\pi F}{2}\right)\sin\left(\frac{n\pi \chi }{2}\right)\sin\left(n\pi S\right).$$

Plugging these expressions into Equations ((4), (5), (6), (7), (8)), we obtain closed‐form expressions of *ω*_g_ and Δ*ω*_±_ as analytic functions on *χ*‐*S* plane. The desired independent control scheme is configured on *χ*‐*S* plane by taking gradients or equi‐level contours of *ω*_g_ and Δ*ω*_±_, as we explain in the next section.

## 3 Rigorous Numerical Analysis

For an exemplary case study, we perform finite‐element method (FEM) calculation of a SiN grating structure embedded in SiO_2_ background. We calculate the key metrics of band‐gap width *ω*_g_ and Δ*ω*_±_ at *k* = 0 on *χ*‐*S* plane, as shown in Figure 2a–c. See the figure caption for fixed design parameters in detail. We compare our simplified model in Section 2 with the FEM calculation. We confirm their quantitative agreement. This agreement of the analytic model for the practically important SiN‐SiO_2_ material platform is one of the key contributions of our approach because the required parametric scans can be obtained without any significant time and computational resource consumption, which are required for the rigorous numerical computation such as the FEM method. In addition, we note that the *χ*‐*S* plane as an efficient parametric space is derived from the closed‐form expressions in Section 2. It is not likely that any fully numerical approach can explicitly reveal such a parametric space in the absence of relevant physical intuition from the analytic theory.

**FIGURE 2:**
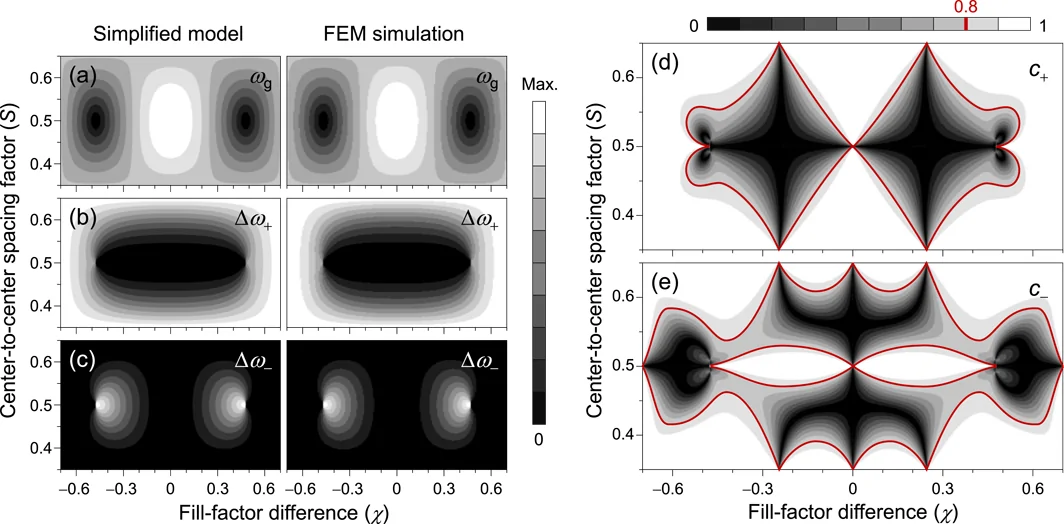
Dependence of key GMR metrics on a design parametric space. (a–c) Band‐gap width *ω*_g_, linewidth at the upper band edge Δ*ω*_+_, and linewidth at the lower band edge Δ*ω*_−_ distributions on *χ*‐*S* space. *χ* and *S* denote the fill‐factor difference and center‐to‐center spacing factor, respectively. We compare the distributions due to our simplified model (left pannels) in Section 2 with rigorous numerical analysis (right pannels) based on the FEM simulation. Maximum values for color density are 9.40 × 10^−3^ for (a), 7.41 × 10^−4^ for (b), and 4.35 × 10^−4^ for (c) in a frequency unit of 2π*c*/Λ. (d) and (e) parametric‐gradient cosine *c*_±_ in Equation (12) that quantifies feasibility of the independent control of *ω*_g_ and Δ*ω*_±_. Regions for |*c*_±_| < 0.8 are identified as the acceptably feasible domains and they are indicated by the red demarcation contours. In this analysis, we assume dual‐ridge SiN grating embedded in SiO_2_ background. Fixed parameters are refractive indices 2.00 for SiN and 1.45 for SiO_2_, ridge height 300 nm, period Λ = 810 nm, and net fill factor *F* = 0.7.

The independent control capability can be quantitatively described by defining parametric‐gradient cosine *c*_±_ such that

(12) $$c_{\pm }=\frac{\nabla \omega_{g}\cdot \nabla \Delta \omega_{\pm }}{\left|\nabla \omega_{g}\right|\left|\nabla \Delta \omega_{\pm }\right|},$$

where the parametric gradient is defined on *χ*‐*S* plane as ∇ = **e**_*χ*_(∂/∂*χ*) + **e**_*S*_(∂/∂*S*). The independent control is infeasible in regions for *c*_±_ ≈ ± 1 while maximally efficient for *c*_±_ ≈ 0. In Figure 2d,e, we show *c*_±_ distributions on *χ*‐*S* plane. Note the regions demarcated by the red contours for the acceptably feasible domains having |*c*_±_| < 0.8.

The independent control is obtained by steering parametric position **p** = (*χ*, *S*) along the equi‐level contour. More specifically, we consider two parametric displacements [Δ**p**]_G_ for *ω*_g_ preserving change and [Δ**p**]_L±_ for Δ*ω*_g±_ preserving change. In Figure 3a,b, we show equi‐level contours of *ω*_g_ and Δ*ω*_±_, and their preserving displacements schematically. Taking the appropriate [Δ**p**]_G_ or [Δ**p**]_L±_ in the feasible regions, we obtain the fully independent control of *ω*_g_ and Δ*ω*_±_.

**FIGURE 3:**
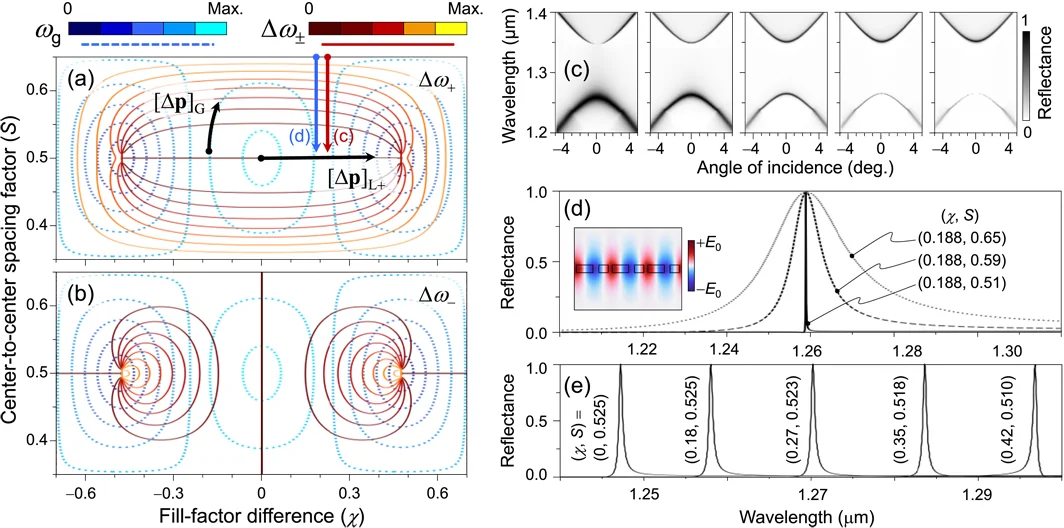
Fully independent parametric control of resonance position and linewidth. (a) and (b) equi‐level contours for *ω*_g_ and Δ*ω*_±_. The blue‐cyan dotted contours in both (a) and (b) indicate *ω*_g_ while the red‐yellow solid contours for Δ*ω*_+_ and Δ*ω*_−_ are separately provided in (a) and (b), respectively. Examples of *ω*_g_‐preserving and Δ*ω*_+_‐preserving displacements are indicated in (a) by black arrows marked with [Δp]_G_ and [Δp]_L+_, respectively. Maximum values for the contour color are identical to those in Figure 2a–c. (c) Angle‐resolved GMR spectra for parametric change along the red arrow marked by “(c)” in (a). The panels are for *p* = (*χ*, *S*) = (0.24, 0.65), (0.24, 0.60), (0.24, 0.55), (0.24, 0.52), and (0.24, 0.50) in the order from left to right. This demonstrates the resonance linewidth change at a constant band gap width of 86 nm. (d) Reflectance spectral profiles under a parametric change along the blue arrow marked by “(d)” in (a). The inset on the left shows relative electric‐field distribution at the resonance peak for (*χ*, *S*) = (0.188, 0.51). It confirms that these GMR features are caused by excitation of TE_0_ guided mode. The peak loci are fixed at 1.259 μm while their linewidth changes from 0.29 to 19 nm. (e) Reflectance spectral profiles under Δ*ω*_+_‐preserving parametric change along [Δp]_L+_. The peak location varies from 1.247 to 1.297 μm while the linewidth is fixed at 0.5 nm.

We note that this approach provides local independent control of target metrics within their restricted and conditional domains, not fully independent control for arbitrary target metric values. Nevertheless, the obtainable target‐metric domains on the accessible parametric regions are large enough to be useful for practical GMR element design in the near‐infrared and telecommunications spectral bands, as demonstrated in the rigorous numerical validation in Figure 3c–e. Band‐gap‐width preserving change of the resonance linewidth is shown in Figure 3c. We apply a *ω*_g_‐preserving displacement starting from (*χ*, *S*) = (0.24, 0.65) and ending at (0.24, 0.50), as indicated by the red arrow with “(c)” mark in Figure 3a. The angle‐resolved transmittance spectra show typical dispersion bands and their linewidth distributions simultaneously. We see that the dispersion curve loci are kept constant while the linewidth significantly changes as desired. Resonance‐position preserving linewidth change is obtained by taking an equi‐frequency displacement indicated by the blue arrow with “(d)” mark in Figure 3a, as shown in Figure 3d. This displacement is close to the *ω*_G_‐preserving displacement for Figure 3c. For this particular parametric displacement, the resonance position of the lower band‐edge GMR remains constant. The associated spectral profile change demonstrates remarkably large linewidth tuning from 22.5 to 0.345 nm while keeping the peak wavelength exactly fixed at 1.259 μm.

The opposite case of resonance location change with a fixed linewidth is also readily possible by taking an appropriate Δ*ω*‐preserving displacement. Figure 3e shows spectral profile change for such a parametric displacement indicated by the black arrow with “[Δ**p**]_L±_” mark in Figure 3a. We confirm that the linewidth is fixed at 0.5 nm while the resonance location varies over 50 nm from 1.247 to 1.297 μm in this demonstration.

Importantly, all these demonstrations are feasible by systematically changing *χ* and *S* without altering other conditions including the net fill factor F, period, and ridge height. In other words, GMR devices for the independent metric controls in Figure 3c–3e can be fabricated on a single uniform thin‐film substrate by a single lithographic process. Therefore, our proposed dual‐ridge compound unit‐cell approach enables remarkably efficient parametric controls of GMR metrics in favor of applications in practice.

## 4 Fermi Arcs in Parameter Space

Resonance‐metric control in periodic lattice structures is immediately related to real and imaginary band structures of corresponding resonance states in general. Real and imaginary band structures respectively describe resonance position and linewidth in *k* space, as also explicitly revealed in Equations ((2), (3), (4), (5), (6)) in our approach here. In the non‐Hermitian physics perspective, particular parametric conditions for real or imaginary bandgap closure are of importance. In our approach, such conditions are expressed by Re[*ω*_G_] = 0 for the real bandgap closure and Im[*ω*_G_] = 0 for the imaginary bandgap closure, respectively, where *ω*_G_ represents complex bandgap which is expressed by the following equation:

(13) $$\omega_{G}=\omega_{+}\left(k=0\right)-\omega_{-}\left(k=0\right)=2\sqrt{m_{+}m_{-}}$$

in our approach. For the real bandgap closure condition of Re(*ω*_G_) = 0, the upper and lower GMR bands touch each other and form a flat, dispersionless segment over a certain *k* range around Γ‐point. This flat segment of merged bands is referred to as real Fermi arc [22]. For the imaginary bandgap closure condition of Im(*ω*_G_) = 0 on the other hand, the bandgap remains opened while the linewidths of the two bands coincide over a certain *k* range around Γ‐point. This uniform linewidth segment is referred to as imaginary Fermi arc in contrast to the real Fermi arc [22]. A real or imaginary Fermi arc terminated by two non‐Hermitian degenerate points known as exceptional point (EP). At EPs, Re[*ω*_G_] = Im[*ω*_G_] = 0 and the eigenstates coalesce into a single state.

The real and imaginary Fermi arcs manifest on a ∞‐shape loop in *χ*‐*S* plane, as shown in Figure 4a. First, the imaginary Fermi arcs are indicated by the blue curves therein. Its condition of Im(*ω*_G_) = 0 results in Δ*ω*_+_ = Δ*ω*_−_., which can be alternatively expressed in terms of Fourier coefficients {*ε*_*n*_}by the following equation:

(14) $$\text{Re}\left(\kappa_{1}\right)=\text{Re}\left(\varepsilon_{2}/{\varepsilon_{1}}^{2}\right)\kappa_{2}.$$

**FIGURE 4:**
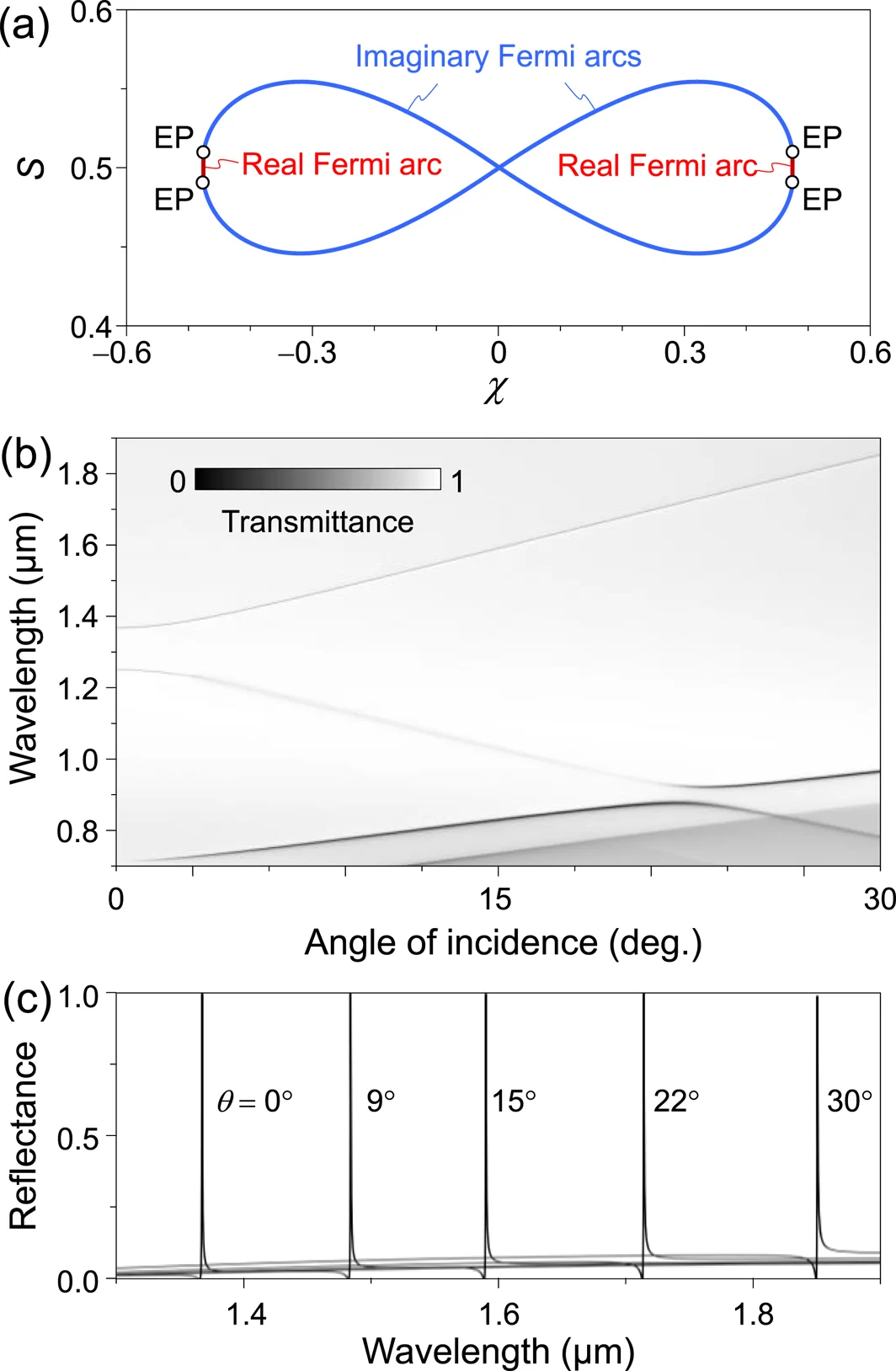
Fermi arcs in GMR spectra. (a) Fermi arcs on *χ*‐*S* plane of our exemplary dual‐ridge unit‐cell structure. (b) Angle‐resolved transmittance spectra on the imaginary Fermi arc at (*χ*, *S*) = (0.1, 0.524). (c) Reflectance spectral profiles at different angles of incidence for (b).

Including Equations (10) and (11), we can readily specify the loop satisfying this condition on *χ*‐*S* plane, as shown in Figure 4a. Note that *κ*_1_ is generally complex‐valued while *κ*_2_ is purely real‐valued in lossless structures [21] and we used it for deriving Equation (14).

On these arcs, the eigenvalue splitting, *ω*_+_ − *ω*_−_ = 2(*m*_+_ *m*_−_ + *vk*)^1/2^ in Equation (3), becomes purely real for *m*_+_ *m*_−_ > −(*vk*)^2^ or otherwise purely imaginary. According to Equation (6) in the purely real splitting case, Δ*ω*_+_ and Δ*ω*_−_ coincide at Im(*δ*) and their explicit *k* dependence vanish. Consequently, we obtain almost uniform linewidth over the entire GMR bands, as shown in Figure 4b for the angle‐resolved transmittance spectra and Figure 4c for the spectral profiles in the reflection. Intriguingly, extremely narrow resonance persists over a wide range of incident angle and wavelength. This property is obtained at any parametric condition on the blue part of the ∞‐shape loop terminated by EPs in Figure 4a.

The remaining piece of the loop indicated by the red segments in Figure 4a connects the EPs on the other side. This segment is referred to as real Fermi arc. On this real Fermi arc, the eigenvalue splitting is purely imaginary because *m*_+_ *m*_−_ + (*vk*)^2^ < 0. Consequently, corresponding GMR dispersion band does not have a band gap, that is, the two band‐edge states appear at a single shared frequency. Instead of being separated in frequency, the two band‐edge states are distinguished in their linewidths. The narrower linewidth state among them dominates physical responses of the structure, potentially creating unique non‐Hermitian dynamics. The real Fermi arc is also known as a critical interface where any parametric displacement passing through it leads to band‐topological phase transition. Therefore, the emergence of the real and imaginary Fermi arcs here motivates further in‐depth study on non‐Hermitian and topological photonics taking advantages of efficient resonance‐metric‐control capability and convenient measurement schemes utilizing inherent leakage radiation as a natural probe that delivers the microscopic effects directly towards the optical far field.

## 5 Conclusion

In conclusion, we have provided a comprehensive description of key GMR metrics in explicit terms of unit‐cell design parameters and proposed a deterministic design method for desired resonance properties. In particular, a dual‐ridge compound unit‐cell structure provides remarkably efficient parametric tuning of dispersion band and linewidth of a GMR in a fully independent manner. Therefore, our results can be immediately used for practical GMR devices such as filters, sensor templates, and surface‐emitting light sources. In addition, we have found out that the dual‐ridge unit‐cell design allows a direct access to emerging physics features associated with unique non‐Hermitian dynamics and topological wave effects such as EP singularities, Fermi arcs, and band‐topological phase transition.

All these benefits originate from the remarkably enhanced design‐parametric controllability on complex‐valued Fourier transform {*ε*_*n*_} which fundamentally determines coupling strengths and phases of elementary diffraction processes. For example, the dual‐ridge compound unit‐cell structures for high index‐contrast conditions demonstrated remarkably efficient spectral‐band engineering of multimode GMR devices including wideband reflectors, polarizers, and bandpass filters [8, 23, 24]. Therefore, it is of great interest to further develop our proposed approach for novel GMR elements involving bound‐states in the continuum, topological localization and beam generation, and many others.

## Author Contributions

**Joong Hyun Pyo:** conceptualization, methodology, investigation, validation, formal analysis, visualization, writing – original draft, writing – review and editing. **Yu Sung Choi:** conceptualization, methodology, investigation, validation, formal analysis, visualization, writing – original draft, writing – review and editing. **Robert Magnusson:** investigation, validation, writing – review and editing. **Jae Woong Yoon:** conceptualization, methodology, software, investigation, validation, formal analysis, supervision, funding acquisition, visualization, project administration, resources, writing – original draft, writing – review and editing.

## Funding

This research was supported by the Leader Researcher Program (Grant NRF‐2019R1A3B2068083).

## Data Availability

The data that support the findings of this study are available from the corresponding author upon reasonable request.
